# Management and outcome in adult intramedullary spinal cord tumours: a 20-year single institution experience

**DOI:** 10.1186/1756-0500-7-908

**Published:** 2014-12-15

**Authors:** Azize Boström, Nina-Christine Kanther, Alexander Grote, Jan Boström

**Affiliations:** Department of Neurosurgery, University of Bonn Medical Center, Sigmund-Freud-Str. 25, Bonn, 53105 Germany

**Keywords:** Intramedullary, Tumours, Outcome, Follow-up, Electrophysiology

## Abstract

**Background:**

Several uncertainties remain concerning the management of intramedullary spinal cord tumours (IMSCTs). These include the timing and extent of resection, its interrelated functional outcome, and the adequate use and timing of radiation therapy and/or chemotherapy. In this retrospective study we report on all adult cases involving IMSCTs treated from 1987 to 2007 in our institution to validate our treatment strategy for IMSCTs. Pre- and post-operative functional performance was classified according to the McCormick scale.

**Results:**

A total of 70 adult cases with IMSCTs consisting of ependymoma (39), astrocytoma (11), carcinoma metastasis (8), haemangioblastoma (5), cavernoma (3) and others (4) were reviewed. Mean age was 46.8 years (range, 18-79 years), and mean follow-up was 4.5 years (range, 1-195 months). The proportion of localisation in descending order was thoracic (36%), cervical (33%), cervicothoracic (19%) and conus region (13%), with 45 gross total resections, 22 partial resections and three biopsies. Surgery-related morbidity with worsening postoperative symptoms occurred immediately in 13 patients (18.6%). The preoperative McCormick grade correlated significantly with the early postoperative grade and the grade at follow-up (χ^2^-test; p = 0.001). None of the patients with preserved intraoperative evoked potentials exhibited significant postoperative deterioration. The degree of resection was correlated with progression-free survival (Duncan test; p = 0.05). Most patients with malignant tumours, namely anaplastic ependymoma (3), astrocytoma (2) or metastatic lesions (5), underwent postoperative radiation therapy. Six patients (one anaplastic ependymoma, two anaplastic astrocytomas and three metastatic lesions) received postoperative chemotherapy.

**Conclusions:**

IMSCTs should be operated on when symptoms are mild. We recommend evoked potential-guided microsurgical total resection of ependymomas and other benign lesions; partial resection or biopsy followed by adjuvant therapy should be confined to patients with high-grade astrocytomas, whereas resection or biopsy with adjuvant therapy is the best option for metastatic lesions.

## Background

Intramedullary spinal cord tumours (IMSCT) are rare lesions and constitute only 4-10% of all primary central nervous system tumours [1, 2]. IMSCTs are less common in adults than in children and constitute 20% and 35%, respectively of all intraspinal tumours [1–4]. The most commonly occurring intramedullary neoplasm is spinal ependymoma followed by glioma and other lesions [2, 5–7]. Treatment strategies in IMSCT have been reported in the past by numerous authors; in the last decade, there seems to have been a general agreement that these lesions need early resection, and whenever feasible complete resection, to achieve a good functional outcome [2, 4, 8].

Nowadays, the microsurgical technique, including the use of an ultrasonic aspirator (Cavitron Ultrasonic Surgical Aspirator [CUSA]), is employed as standard. Many studies have recommended the routine use of electrophysiological monitoring, mostly somatosensory-evoked potentials (SSEP), although in some cases motor-evoked potentials (MEP) are also used; however, only a few studies have evaluated their effective impact [9–12]. In some reports, the study period dates back to the non-microsurgical and non-magnetic resonance imaging (MRI) era, providing the opportunity to evaluate a long mean follow-up period and a resultant increase in the statistical power. However, this makes it impossible to exclude biases from changes in the treatment strategy, diagnostic imaging technologies and surgical techniques in the long-term. The number of reported cases ranges between 34 and 202 [2–4, 8].

The purpose of the present study was to retrospectively review the results regarding the surgical treatment of IMSCT at our hospital during the last 20 years, to elucidate the problems associated with surgery and to validate the treatment strategy for these tumours.

## Methods

All adult patients who underwent surgical therapy for an IMSCT at our institution between 1987 and 2007 were reviewed for this study; only children with an IMSCT were not included. Patient charts, and surgical and histological reports were analysed. In those patients with no follow-up data in the charts, a standardised telephone interview was performed. Two patients were not available for a telephone interview.

Standard therapy consisted of a complete resection involving a microsurgical technique using CUSA whenever feasible. Tumours were operated on using standard dorsal approaches as indicated by the location of the lesions (classification of the location: C0-C7 = cervical, TH1-12 = thoracic and L1-5(6) = lumbar with conus; tumours involving cervical segment(s) and thoracic segment(s) were classified as cervicothoracic and tumours involving thoracic and lumbar segments were classified as thoraco-lumbar). Regularly, it was a laminectomy that was performed. Only the multi-level cases underwent surgery using laminoplasty. There was no case involving primary dorsal fusion. A sitting position was favoured if the tumour was localised in the upper cervical region, and a prone position was favoured if the tumour was localised in the lower cervical, thoracic or lumbar spine. After midline dural incision over the dorsal surface of the spinal cord, followed by lateral dural sutures, a midline myelotomy was carried out and used as a natural plane of dissection to avoid injury to the axonal tracts.

Recognition of the accurate position of the midline prior to myelotomy is essential in minimising neurological defects. We relied on the identification of the posterior spinal arteries in the healthy spinal cord, located proximally and distally to the tumour, to estimate the myelotomy incision site.

In our hospital, intraoperative monitoring was implemented early in 1989; hence, this technology was available for 18 out of the 20 years of the study period. In all cases, the tumour was obtained for histopathological examination and classified according to the World Health Organisation (WHO) classification at the same institution whenever feasible (Reference Centre for Brain Tumours). Each patient’s neurological status was recorded using the McCormick classification scheme (i.e. ranging from Grade I [neurologically intact] to Grade IV [severe functional disability]) [5].

### Statistical analysis

Tumour extension and localisation, preoperative neurological status, WHO grade and electrophysiological results were correlated with neurological outcome postoperatively and at follow-up. Progression-free survival (PFS) was estimated using the Kaplan-Meier technique. The χ^2^-test for independence, and univariate and multivariate analysis were employed for statistical evaluation using Excel (Microsoft, Seattle, WA, USA) and SPSS (SPSS Inc., Chicago, IL, USA) software.

Pertinent clinical data and follow-up information were collected by means of chart review and telephone interviews, as required. The clinical data were collected after informed consent to conduct the telephone interviews. Consent to publish was obtained from patients in accordance with the tenets of the declaration of Helsinki, and after approval of the study by the Ethics Committee of the Medical Faculty of the University of Bonn.

## Results

**Figure 1:**
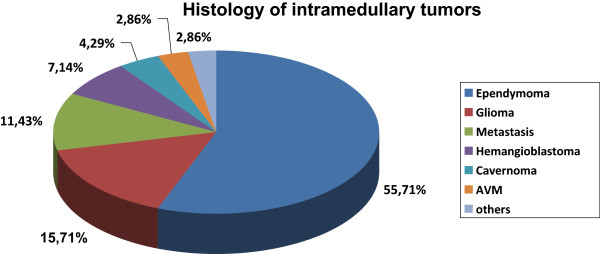
**Histology of intramedullary tumours.** Incidence of histologies of intramedullary tumours over a 20 year period (1987-2007).

**Figure 2:**
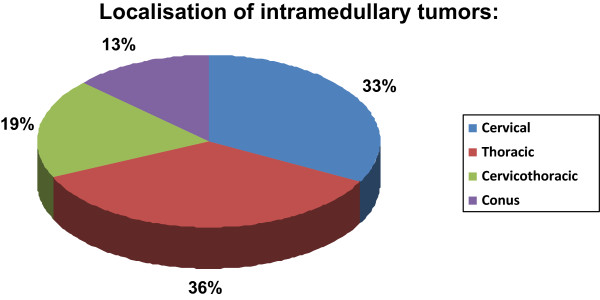
**Localisation of intramedullary tumours.** Incidence of localisations of intramedullary tumours over a 20 year period (1987-2007).

A total of 70 adult patients (37 men and 33 women) underwent primary surgical therapy for IMSCT at our institution; five children with IMSCT were excluded from in this study. The patients ranged in age from 18 to 79 years (mean 46.8 years) with a median follow-up of 55 (range, 1-195) months. Only one patient with an ependymoma had undergone a previous resection. Histological diagnosis revealed ependymoma (n = 39; including one subependymoma), 11 glial tumours (pilocytic astrocytoma WHO Grade I [n = 5]; glioma WHO Grade II [n = 3]; pleomorphic xanthoastrocytoma [n = 1]; anaplastic astrocytoma WHO Grade III [n = 3]), carcinoma metastasis (n = 8), haemangioblastoma (n = 5), cavernoma (n = 3), arteriovenous malformation (AVM; n = 2) and other histological diagnoses [n = 2]) (Figure 1). The most frequently involved localisation was in the thoracic region (36%), followed by the cervical region (33%), the cervicothoracic region (19%) and the thoraco-lumbar (with conus) region (13%) (Figure 2). One case demonstrated holospinal involvement. We performed 45 gross total resections, 22 partial resections and three biopsies.

Preoperative SSEP/MEP monitoring was performed in 41 cases (58%) and intraoperative monitoring in 24 cases (35%; among them there were 16 SSEP and/or 19 MEP investigations). Postoperative SSEP/MEP monitoring for follow-up was performed in 24 cases (24%).

### Neurological outcome

**Figure 3:**
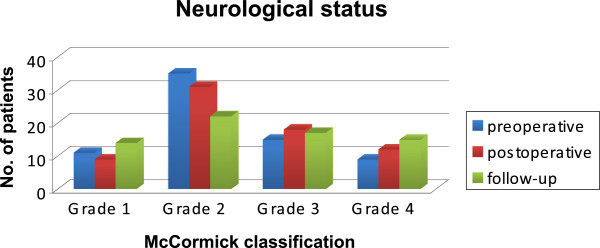
**Neurological status.** Development of neurological status according to the McCormick classification.

The results concerning the McCormick graduation pre- and post-operatively (before discharge) and at last follow-up are presented in Figure 3 and Tables 1 and 2. No surgery-related mortality was encountered. An immediate improvement after surgery was achieved in five (7.1%) patients. Surgery-related morbidity with a worsening of symptoms immediately after the surgical procedure occurred in 13 (18.6%) patients. In three patients, the McCormick grade improved during follow-up to the same grade as that prior to surgery. In total, the overall surgery-related permanent morbidity was 14.3%. It is noticeable that in 8/10 (80%) cases with permanent deterioration, the resection was incomplete. Nearly all patients with a high grade tumour (WHO Grade III ependymoma or astrocytoma, and all metastatic lesions) were worse on discharge or at last follow-up because of progression or the presence of a recurrent tumour. Most patients with benign or vascular lesions (cavernoma, AVM, haemangioblastoma) remained stable post-operatively and at follow-up (for details see section entitled *Separate analysis of different pathologies*).

**Table 1:** **Neurological status according to McCormick classification postoperatively significantly depending on the preoperative neurological condition (chi-square test, p = 0.001)**

McCormick grade postoperative	McCormick grade preoperative			
	I	II	III	IV
	(n = 11)	(n = 35)	(n = 15)	(n = 9)
**I**	7 (63,6%)	2 (5,7%)	0	0
**II**	3 (27,3%)	26 (74,3%)	2 (13,3%)	0
**III**	1 (9,1%)	5 (14,3%)	11 (73,3%)	1 (11,1%)
**IV**	0	2 (5,7%)	2 (13,3%)	8 (88,9%)

**Table 2:** **Neurological status according to McCormick classification at follow-up significantly depending on the preoperative neurological condition (chi-square test, p = 0.001)**

McCormick grade at follow-up	McCormick grade preoperative			
	I	II	III	IV
	(n = 11)	(n = 34)	(n = 15)	(n = 8)
**I**	7 (63,6%)	7 (20,6%)	0	0
**II**	3 (27,3%)	16 (47,1%)	3 (20,0%)	0
**III**	1 (9,1%)	8 (23,5 %)	6 (40,0%)	2 (25,0%)
**IV**	0	3 (8,8%)	3 (20,0%)	6 (75,0%)

### Adjuvant therapy

Subsequent adjuvant therapy, which consisted of radiation therapy and/or chemotherapy, was only administered to patients with neuroepithelial WHO Grade III tumours (n = 5) and in all but one patient with metastases including an esthesioneuroblastoma metastasis (n = 7). For details see section entitled *Separate analysis of different pathologies*.

### Statistical analysis

Advancing age (>60 years) was not found to be a statistically relevant prognostic factor regarding PFS in our series (p > 0.05). Age and gender had no significant effect on the post-operative neurological outcome or on neurological outcome at follow-up (p > 0.05). In addition, the WHO grade (if applicable) had no significant effect on the neurological outcome at follow- up in our series (p > 0.05). The degree of resection (biopsy vs. total resection) was correlated with PFS (Duncan test, p = 0.014). It was also found that pre-operative status (McCormick grade) was significantly correlated with the early post-operative grade and with the grade at follow-up (χ^2^-test, p = 0.001) (Tables 1 and 2).

### Separate analysis of different pathologies

#### Ependymoma (n = 39)

Total resection was achieved in 30 of the 39 cases of ependymoma (79%). In the remaining cases, only subtotal resection could be performed because the boundary between the tumour and the spinal cord was indistinct (n = 8), or the amplitude of the intraoperative spinal evoked potential decreased frequently (n = 1).

Most tumours were WHO Grade II (n = 31); in one case, a primary anaplastic ependymoma WHO Grade III was diagnosed, while the first tumour recurrence in another case was WHO Grade III. The remaining six tumours were WHO grade I. Both anaplastic tumours received radiation therapy. One patient with a WHO Grade II ependymoma exhibited further recurrent tumour at 4 years after, which was five levels below the initial tumour localisation. In this case, a neoplastic meningiosis was diagnosed and the patient underwent additional chemotherapy consisting of the intrathecal application of etoposid (VP16) and salvage chemotherapy with carboplatin, cyclophosphamide and vincristine.

Tumour recurrence after complete resection (n = 2) or tumour progress after subtotal removal (n = 4) of the ependymomas was encountered in 6/39 cases (15%). In five patients, recurrence occurred at the former tumour level, and at multiple levels in one patient. Recurrent tumours of WHO Grade I were diagnosed between 36 and 59 months (mean 46 months) after treatment; interestingly, all were ependymoma of the myxopapillary subtype. In patients with WHO Grade II tumours, the recurrences occurred between 5 and 24 months (mean 14 months) after the first operation. In four cases, functional improvement was obtained after complete resection. Deterioration was observed post-operatively in eight patients. In three cases, there was a subtotal resection (3 of 9 incomplete resections = 33%). Deterioration was observed in only five of the 30 completely resected tumours (17%).

A not significant but potential prognostic factor regarding ependymomas appears to be tumour localisation. An investigation concerning the impact of the level of tumour localisation on treatment outcome revealed that in five out of 20 patients (25%) with thoracic involvement, the outcome was worse than was the case for cervical or conus/lumbar localised tumours (15% [2/13] and 17% [1/6], respectively). Improvement was observed in one of the cervical and one of the cases with conus involvement, whereas only three of the thoracic cases showed improvement.

In summary, the prognosis of ependymomas appears to be favourable, with 36 patients (89.5%) still alive and in good condition at the last follow-up. The functional outcome in ependymomas was favourable if preoperative paralysis was mild.

#### Glioma (n = 11)

Total resection was achieved in only one of eight cases of low grade glioma, and a subtotal resection (>90%) was achieved in another case. A partial resection was performed in four patients because the boundary between the tumour and the spinal cord was indistinct. Biopsy alone was performed in the remaining two patients, because of the location and multilevel extension in one cervical (four levels) and one cervicothoracic (15 levels) case. However, in the high-grade group, no total resection or subtotal resection was performed; here only partial resection (two cases) or biopsy (one case) was performed.

One incompletely resected WHO Grade I astrocytoma recurred at 3 years after treatment as a Grade III tumour, and the patient received both radiation therapy and chemotherapy consisting of nimustine (ACNU). Both patients with WHO Grade III primary anaplastic astrocytomas received chemotherapy consisting of procarbacin, carmustin and vincristine (standard PCV scheme).

The functional outcomes were poorer in the astrocytoma than in the ependymoma cases; post-operative improvement of paralysis was found only in one patient at last follow-up. Nine of 11 patients (82%) were neurologically stable and two patients were worse; one had a thoracic low-grade tumour which was spreading over 10 levels with partial resection and one was found to have a cervicothoracic high-grade glioma after biopsy. Of the 11 glioma patients, three patients in the low grade group had died at the time of the final survey, but there were two 5-year survivors in the high grade group. Because of the small number of cases, assessment of the impact of the differences in the surgical procedures and the impact of the level of the tumour localisation on the prognosis did not reveal any statistical significance.

#### Metastatic lesions (n = 8)

Total resection was possible in 50% of the cases. Adjuvant therapy in the form of radiation therapy was administered to five out of the eight patients (total dose, 30–40 Gray [Gy]), two patients received additional chemotherapy and one patient received palliative chemotherapy alone. One patient died before the planned adjuvant therapy. In addition, five of the eight cases arose from primary lung cancer, with small cell carcinoma being the most common subtype; one metastasis originated from renal cell carcinoma (n = 1), one case was a filia of an esthesioneuroblastoma, and there was one metastasis of a cancer of unknown primary (CUP) with histologically proven adenocarcinoma.

All five lung cancer patients died within 6 months of treatment; the patient with renal cell carcinoma experienced a symptom-free interval of 13 months, the CUP patient had a follow-up period of 8 months and the patient with the esthesioneuroblastoma was recurrence free for 4 months. At post-operative evaluation, one patient had improved and one had deteriorated; all of the other patients were functionally similar. At the last follow-up, the three surviving patients demonstrated deterioration caused by disease progression.

#### Haemangioblastoma (n = 5)

The strategy for the microsurgical removal of haemangioblastoma was somewhat different from other IMSCTs. Our strategy was to coagulate the dominant arterial feeders at the entrance of the tumour (in most cases these were identified by additional spinal angiography) by bipolar coagulation involving low power first, followed by coagulation and shrinkage of the tumour and lastly coagulation of the venous drainage. Finally, the tumour was carefully excised en bloc. Total resection was possible in four of five patients; in the remaining patient only partial resection could be performed because the intra-operative spinal evoked potentials were lost. In this case and in one other case, von Hippel-Lindau disease was diagnosed. Only the one patient who underwent partial resection showed a relevant neurological deficit pre- and post-operatively; in all of the other patients, pre-operative paralysis was mild and their functional outcomes were even better than those who had ependymomas, with one postoperative improvement in one patient and a stable condition in all of the other patients. The patient who underwent an initial partial resection developed two recurrences together with further neurological deterioration.

#### Vascular lesions (n = 5)

Cavernomas and AVMs are very rare and extremely variable (in terms of size, location and the vessels involved), usually making the interpretation of surgical results case sensitive. We treated three patients with cavernoma and two patients with an AVM; all of these intramedullary vascular lesions could be resected *in toto*. In two patients with cervical cavernomas, a worsening from McCormick Grade II to III at post-operative evaluation was noted; both were stable at the last follow-up and a third case with a medullo-cervical cavernoma demonstrated a late deterioration at the last follow-up. In the AVM patients, there was one worsening post-operative (conus) case and one improvement at last follow-up (cervical AVM with incomplete embolisation pre-operatively).

#### Other histological diagnoses (two cases)

One case was a 28 year old male patient who had a thoracolumbar intramedullary dermoid cyst with associated spina bifida occulta at level L5/S1. At the immediate post-operative evaluation, the patient remained neurologically stable; at the late follow-up (4.5 years post-operatively) the patient exhibited a significant neurological deterioration (McCormick Grade IV), which was most likely associated with a tethered cord. The other case, a 73 year-old female who had severe functional disabilities, was a patient who had a spontaneous cervical haematoma caused by marcumar therapy, without histological proof of any tumour or vascular malformation before surgery, and who did not show neurological improvement post-operatively (McCormick Grade IV).

## Discussion

The management of intramedullary spinal cord tumours has been influenced by some drastic improvements in treatment during the last decades. Advances in imaging and surgical techniques have led to the removal of many tumours with a high success rate and low morbidity [2, 10, 13]. However, the relative rarity of these tumours makes the accumulation of data from large patient series difficult. In many situations, there is still an incomplete knowledge base regarding the optimal management of IMSCT. Some published series providing long-term follow-up data have included paediatric patients or patients treated long before the advent of the microsurgical and MRI-era [3, 4, 13, 14]. The present report details functional and oncological outcomes, together with electrophysiological data from adult patients with IMSCT operated on solely in the microsurgery and MRI-era from 1987–2007 at one single institution. Although we have exclusively analysed adult cases in the microsurgical and MRI-era, the number of patients (n = 70) enrolled in our study and the mean follow-up period (55 months) are comparable with former reports [3, 8, 15].

In a series of 202 cases involving long-term follow-up of IMSCT, Raco et al. [4] found a number of potential determinant predictors of a good outcome after surgery, including histological type of lesion, complete removal of the lesion and satisfactory neurological status before surgery. These findings are in line with ours. However, the WHO grade had no significant effect on the neurological outcome at follow-up in our series. Raco et al. [4] presented a large case series, but included patients over a longer period (1972– 2003) than was the case in our study; therefore, many cases were treated well before the MRI and microsurgery-era. We excluded all of those cases to avoid any bias regarding changes in surgical and diagnostic quality.

In 2005, Sandalcioglu et al. [8] reported their experience with 78 patients between 1990 and 2000. In their series, the strongest predicting factor of functional outcome was again the preoperative neurological condition, beyond the histological differentiation of IMSCT. Although there was no difference in therapeutic outcome with respect to age and tumour extension, thoracic IMSCT was found to harbour an increased risk of postoperative surgical morbidity. In our series, higher morbidity was associated with surgical removal of multilevel thoracic lesions without reaching statistical significance. Thoracic tumours spanning several levels may require extensive dissection and manipulation of the spinal cord to expose the tumour, with an increased risk of postoperative surgical morbidity.

In 2009, Matsuyama et al. [13] reported on 106 patients operated on between 1997 and 2007; the mean follow-up period was 7.3 years. The age range was 6-75 years; therefore, in this series, infantile cases were included. Because the natural history of most infantile cases differs relative to the adult cases, we also excluded infantile cases in our series. Again, the postoperative ambulatory ability was excellent in patients with a good preoperative neurological status. Total excision in patients with good ambulation was associated with a good prognosis for post-operative mobility. The rate of postoperative deterioration was 31% [13], which was relatively high compared with our series with a rate of early post-operative deterioration of 18.6%. We can confirm that individuals with mild-to-moderate deficits often improve significantly following surgical removal of the tumour, while those with advanced neurological compromise generally exhibit no worthwhile improvement. In our opinion, this emphasises the need for early intervention and close follow-up.

Some additional general conclusions can be drawn from our data, in combination with the relevant literature. Although the value of total excision of ependymomas is widely accepted [16–19], the value of radical resection of astrocytomas is less certain. If an ill-defined plane is present, the risk-to-benefit ratio for aggressive removal is not clear. Currently, no satisfactory modality is available for the treatment of non-resectable and malignant astrocytomas. Fortunately, anaplastic astrocytoma or glioblastoma are rare. Surgical therapy alone does not improve the usually dismal disease course; therefore, adjuvant radiation therapy and/or chemotherapy are options [6, 14, 20–26]. Data are available which suggest that surgically excised ependymomas do not need to undergo subsequent radiation therapy [16–19]. However, the optimal management of residual or recurrent tumour has not been determined. Whether or not repeat excision, watchful waiting or radiation therapy is the best choice requires further clarification [1, 27].

Radiation therapy involving neuroepithelial tumours may be useful for residual tumour after surgery and recurrent tumour, but controversy exists regarding this treatment [18, 28, 29]. This modality may also be the primary treatment for inoperable tumours and aggressive lesions such as anaplastic astrocytomas and glioblastomas; however, currently no radio-oncologist should undertake radiation therapy without a tissue diagnosis. Some studies have reported reduced local failure rates when a total radiation dose of 50 Gy was administered [22, 30]. Modern techniques like image-guided radiotherapy or stereotactic radiosurgery can ensure the delivery of a therapeutically effective dose to the tumour while spearing the healthy surrounding tissue; these techniques may have a role to play in management in the future, in a similar manner to their current role in the management of intracranial tumours [31–33].

Chemotherapy is still considered experimental in the treatment of adult spinal cord tumours; a number of protocols are undergoing examination, primarily involving childhood astrocytomas [34–36]. We have used standard chemotherapy protocols for cerebral gliomas (PCV and ACNU) for malignant neuroepithelial tumours in our series. We can report two long-term survivors with anaplastic astrocytoma after a follow-up time of >5 years who are still alive, and have received radiation therapy (total doses of 45 and 50.4 Gy) and chemotherapy (ACNU and PCV).

Total removal of the neoplasm, with preservation of neurologic function, is the goal in most cases. Monitoring spinal cord function using intraoperative electrophysiology modalities, such as SSEP and motor-evoked potentials (MEP), may reassure the surgeon during the procedure and perhaps lead to improved outcomes [12]. None of our patients with preserved intra-operative MEP/SSEP exhibited immediate significant postoperative deterioration. In our series, the fact that intraoperative electrophysiology was used in only 35% of all cases, and monitoring was not applicable in 12% of the cases because of technical problems, attracted our attention; thus it is remarkable that our study outcome is comparable with those of other studies with the use of much more extensive intraoperative monitoring [9, 10, 13].

### Study limitations

Because this study involved a single centre, the data presented are not representative and the results cannot be generalised. However, the study can still be of value when viewed as a sample study submitted by a major neurosurgery department. Vascular lesions are very rare and extremely variable (in terms of size, location and vessel involvement), usually making the interpretation of surgical results case sensitive. Consequently, such rare lesions should be further investigated in multi-centre series.

## Conclusions

Intramedullary ependymomas should be operated on when symptoms are mild. Early surgery can achieve an excellent outcome. We recommend evoked potential-guided aggressive microsurgical resection of most ependymomas and other benign lesions, because these lesions regularly showed excellent functional outcome and no tumour recurrence. Partial resection or biopsy followed by radiation therapy should be confined to patients with high-grade astrocytomas. Biopsy or partial resection and adjuvant therapy involving radiation therapy and/or chemotherapy, depending on the primary tumour type and site, is the preferred option regarding metastatic lesions.

## Consent

Clinical data was collected after written informed consent to publish details included within this research article was obtained from all of the patients in accordance with the tenets of the declaration of Helsinki and after approval by the Ethics Committee of the Medical Faculty of the University of Bonn. An additional consent from the next of kin of deceased patients here was not necessary because each patient has signed his consent on admission to our hospital, that his data may be scientifically evaluated and published.

## Abbreviations

AVM: Arteriovenous malformation
CUSA: Cavitron ultrasonic surgical aspirator
CUP: Cancer of unknown primary
IMSCT: Intramedullary spinal cord tumour
MEP: Motor-evoked potentials
PFS: Progression-free survival
SSEP: Somotosensory-evoked potentials
WHO: World Health Organisation
PCV: Procarbazine carmustine vincristine
ACNU: Nimustine
MRI: Magnetic resonance imaging
Gy: Gray.
